# Single-cell genome-wide bisulfite sequencing uncovers extensive heterogeneity in the mouse liver methylome

**DOI:** 10.1186/s13059-016-1011-3

**Published:** 2016-07-05

**Authors:** Silvia Gravina, Xiao Dong, Bo Yu, Jan Vijg

**Affiliations:** Department of Genetics, Albert Einstein College of Medicine, Michael F. Price Center, 1301 Morris Park Avenue, Bronx, NY 10461 USA; Department of Obstetrics & Gynecology and Women’s Health, Albert Einstein College of Medicine, Bronx, NY 10461 USA; Present address: Illumina Inc, San Diego, CA 92122 USA; Present address: Department of Obstetrics and Gynecology, University of Washington, Seattle, WA USA

**Keywords:** Single-cell epigenomics, Epivariations, Epigenetic instability, Single-cell DNA methylomics, Aging

## Abstract

**Background:**

Transmission fidelity of CpG DNA methylation patterns is not foolproof, with error rates from less than 1 to well over 10 % per CpG site, dependent on preservation of the methylated or unmethylated state and the type of sequence. This suggests a fairly high chance of errors. However, the consequences of such errors in terms of cell-to-cell variation have never been demonstrated by experimentally measuring intra-tissue heterogeneity in an adult organism.

**Results:**

We employ single-cell DNA methylomics to analyze heterogeneity of genome-wide 5-methylcytosine (5mC) patterns within mouse liver. Our results indicate a surprisingly high level of heterogeneity, corresponding to an average epivariation frequency of approximately 3.3 %, with regions containing H3K4me1 being the most variable and promoters and CpG islands the most stable. Our data also indicate that the level of 5mC heterogeneity is dependent on genomic features. We find that non-functional sites such as repeat elements and introns are mostly unstable and potentially functional sites such as gene promoters are mostly stable.

**Conclusions:**

By employing a protocol for whole-genome bisulfite sequencing of single cells, we show that the liver epigenome is highly unstable with an epivariation frequency in DNA methylation patterns of at least two orders of magnitude higher than somatic mutation frequencies.

**Electronic supplementary material:**

The online version of this article (doi:10.1186/s13059-016-1011-3) contains supplementary material, which is available to authorized users.

## Background

Transmission fidelity of CpG DNA methylation (5mC) patterns is not foolproof, with error rates from less than 1 to well over 10 % per CpG site, dependent on preservation of the methylated or unmethylated state and the type of sequence [1, 2]. This suggests a fairly high chance of errors. Indeed, while the numerous cellular identities in complex metazoa are shaped by epigenetic regulation, there is a lack of information as to the stability of epigenetic marks, such as DNA methylation, in differentiated cell types during development and aging. However, the consequences of such errors as well as regulated variation, in terms of increasing 5mC variance between a single cell and the bulk cell population, here termed “epivariation”, in tissues of an adult organism have never been demonstrated experimentally.

Using a single-cell bisulfite PCR-based approach, we have recently shown that, within a few selected gene promoter regions of mouse hepatocytes, the frequency of epivariations due to erroneous methylation or demethylation of a CpG site is indeed quite high, i.e., between 1.6 % for methylating epivariations and 2.7 % for demethylating epivariations [3]. This finding prompted us to directly test for epivariation in DNA methylation across the entire genome in mouse liver hepatocytes. Our results indicate a level of epimosaicism in adult mouse liver that is very high, corresponding to an average epivariation frequency of 3.3 %. Interestingly, the level of 5mC instability was found to depend on specific genomic features, with promoters and CpG islands being the most stable and non-functional regions the most unstable. Such heterogeneity could be responsible, at least in part, for the remarkably large intrinsic gene expression variability observed among hepatocytes in mammalian liver [4].

## Results and discussion

### Single-cell whole-genome bisulfite sequencing accurately reports genome-wide 5mC patterns

In order to experimentally measure intra-tissue liver heterogeneity, diploid hepatocytes were isolated from six mice, three 4-months old and three 26-months old, by liver perfusion and sorted using fluorescence-activated cell sorting (FACS) into PCR tubes after Hoechst 33342 staining. A total of 21 hepatocytes (at least two cells per animal) were subjected to bisulfite treatment followed by whole-genome library preparation and sequencing on an Illumina HiSeq 2500 with 100-base, single-end reads. For each animal we also performed whole-genome bisulfite sequencing (WGBS) of DNA from bulk hepatocytes. For comparison, we also sequenced libraries generated from five manually picked individual mouse embryonic fibroblasts, as well as DNA from two bulk fibroblast cell populations.

**Fig. 1:**
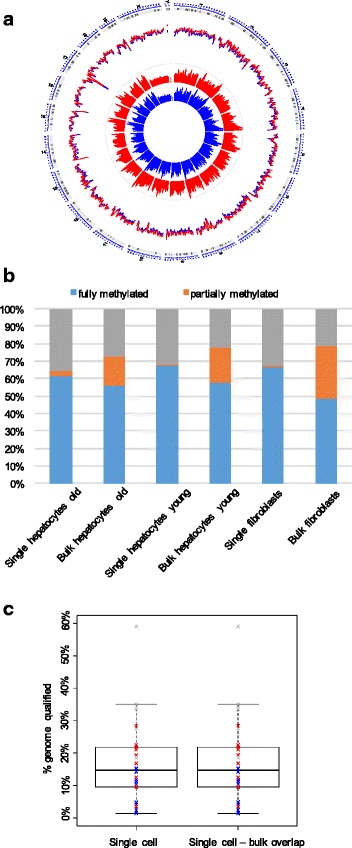
Global methylation and coverage of single-cell WGBS. **a** Genome wide 5mC levels and coverage in single fibroblasts (*blue*) and hepatocytes (*red*). From outside to inside, the *first layer* represents 5mC level, the *second layer* coverage at each CpG site. 5mC levels and coverage were averaged among cells from each group and estimated using 1-Mb non-overlapping sliding windows. **b** Global 5mC levels at CpG sites for single cells and bulk for the two cell types and two age groups. **c** Percentage of genomic 3-kb windows containing at least 5 CpG sites in single hepatocytes and fibroblasts. Virtually all qualified windows in the single cells were found to overlap with their bulk samples. *Grey*, fibroblasts; *blue*, young hepatocytes; *red*, old hepatocytes

On average, 17 million reads were mapped for each single hepatocyte (109 million for the bulk), corresponding to a mapping efficiency of 32.3 % for the single cells and 55.9 % for the bulk (Additional file 1: Table S1). The somewhat lower mapping efficiency of the single-cell DNA may be due to reduced complexity of the DNA amplified from single cells compared with the bulk and was also found by Smallwood et al. [5]. Mapped reads appeared as randomly distributed across the genome, providing information on all genomic features, covering 2.2 million CpG sites on average for the single cells and 21.6 million on average for the bulk DNAs (Additional file 1: Figure S1 and Table S1). Coverage was distinctively lower for the single cells than for the bulk DNA, in agreement with data from others [5] (Fig. 1a). Bisulfite conversion efficiency was 98 % or higher, as assessed by analysis of non-CpG methylation (Additional file 1: Table S1). Additional file 1: Table S2 compares these methodological specifics with two previously published protocols for single-cell methylomics [5, 6], showing that performances of all three methods are fairly similar (Additional file 1: Table S2).

While the vast majority of CpG sites were either methylated or unmethylated, a small fraction was found to show partial methylation (1.07 ± 0.98 %; Fig. 1b; Additional file 1: Figure S2). Interestingly, the fraction of partially methylated sites was consistently smaller in single cells compared with the bulk (Fig. 1b). While in bulk cell populations partially methylated sites most likely reflect amplification from multiple alleles (from many cells), in single cells amplification is only from two alleles and, in practice, due to the low coverage in most cases, from only one. This is the most likely explanation for the very small fraction of partially methylated sites in single cells. This is in keeping with the slightly higher fraction of partial methylation in the manually selected control fibroblasts, coverage of which was significantly higher than the hepatocytes (due to better single-cell DNA quality in these cells, which had not gone through enzymatic isolation and cell sorting; Fig. 1b).

**Fig. 2:**
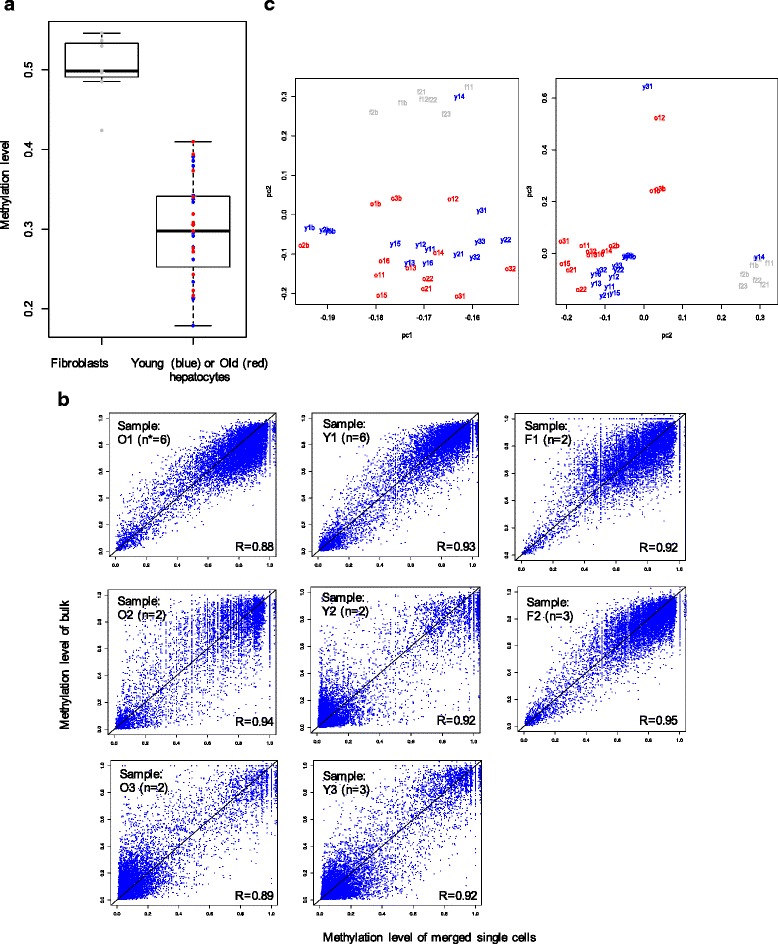
Single-cell WGBS is an accurate and reproducible method for genome-wide 5mC analysis. **a** 5mC promoter methylation status of 58 liver-specific genes. **b** Merged single cells have the same methylation pattern as their corresponding bulk. Each comparison is based on 10,000 randomly chosen 3-kb windows indicates the number of single cells sequenced. **c** Principal component analysis of single cells and bulk shows separate clustering of fibroblasts and hepatocytes (both panels) and hepatocytes from old and hepatocytes from young mice

To analyze 5mC patterns in the isolated hepatocytes, we subdivided the genome in 3-kb sliding windows with a step size of 600 bp and determined the weighted average of 5mC in each window as described in Additional file 1: Supplemental Experimental Procedures. Only windows containing at least 5 CpG sites were used for the analysis (Fig. 1c). Before proceeding with an in-depth analysis of 5mC heterogeneity, we verified the accuracy of our single-cell procedure in faithfully reporting correct DNA methylation status using two approaches. First, we tested if the DNA methylation patterns obtained for the studied single cells were indeed specific for hepatocytes. Because 5mC promoter status is generally inversely correlated with gene expression status [7–9], we used a set of 58 liver-specific genes identified through multi-tissue RNA-seq analyses by Lin. et al [10]. As those genes are specifically expressed in the liver, we expected their promoters to be generally hypomethylated. Indeed, our results show that the average 5mC level of promoters of these liver-specific genes in all single hepatocytes was very similar to that in the bulk hepatocytes and significantly lower (*p* < 0.001, one-tailed *t*-test) than that in the promoters of these same genes in fibroblasts (Fig. 2a).

Second, we merged methylation patterns of single hepatocytes as well as fibroblasts and compared this with their corresponding bulk patterns. Similar methylation levels of the merged and the bulk were observed for every window (Fig. 2b). Principle component analysis (PCA) revealed that single cell and bulk cluster according to cell type and age (Fig. 2c). In both cell types the clustering was affected by sequencing coverage, as expected because of the much higher coverage of the bulk samples (Fig. 2c; Additional file 1: Figure S1). We noticed that one young hepatocyte, “y14”, clustered with fibroblasts in PCA, although it has similar promoter methylation patterns to liver-specific genes. This may reflect the diversity and heterogeneity of the hepatocyte population. Based on the promoter methylation patterns and PCA clustering, we conclude that our single-cell DNA methylomics protocol correctly identified cell type-specific DNA methylation patterns.

### 5mC heterogeneity in liver is high and dependent on sequence feature

**Fig. 3:**
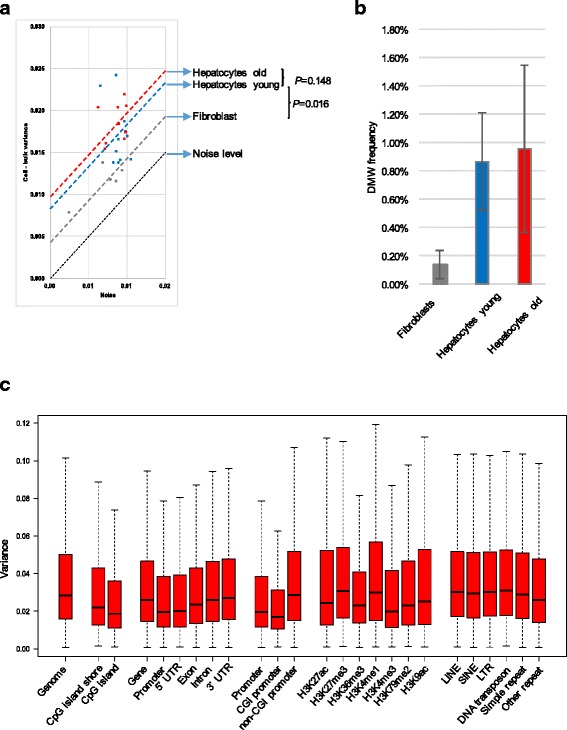
5mC heterogeneity. **a** Global heterogeneity per cell. Variance value was used to quantify the difference between a cell and its bulk across windows. Raw variance (*x-axis*) and noise (*y-axis*) estimated from downsampling bulk to single-cell equivalent were plotted. To test significance of difference in mean variance among groups, *P* values were obtained by using permutation tests of randomly resampled samples into the two groups for comparison. **b** Number of differentially methylated windows in fibroblasts and hepatocytes from young and old mice. Differentially methylated window (DMW) frequency was significantly higher in hepatocytes than in fibroblasts (*P* < 0.001, two-tailed *t*-test). The slightly higher DMW frequency in hepatocytes from aged mice was not significant. **c** 5mC heterogeneity in liver is highly dependent on sequence feature. *CGI* CpG island, *LINE* long interspersed nuclear element, *LTR* long terminal repeat, *SINE* short interspersed nuclear element, *UTR* untranslated region

To quantitatively analyze 5mC heterogeneity among the single hepatocytes, we compared the average 5mC content of the 3-kb sliding windows overlapping between each single hepatocyte and its bulk population and calculated the variance between each cell and the bulk from which it was derived (Fig. 3a; Additional file 1: Supplemental Experimental Procedures). To control for possible artifacts caused by sequencing depth variation, we also calculated the variance between the bulk and artificial cells, simulated by downsampling of the bulk itself to a single-cell sequencing depth. This “noise” (y-axis, Fig. 3a), which was between 53.3 ± 13.0 % of the actual variations between the real cells and the bulk, was then subtracted from the raw variance values (x-axis, Fig. 3a). The results confirm a significant level of cell-to-cell variation in 5mC across the genome. As expected, heterogeneity was significantly higher among hepatocytes compared with the five fibroblasts included as control cells (*P* = 0.016, one-tailed permutation test on the mean variance of each group). As these cells had been taken from the same plate, both the number of cell divisions and chronological time between them was much shorter than for the hepatocytes, each of which went through the process of development and aging, with ample opportunity to undergo epivariation.

Due to the high level of 5mC heterogeneity, both in young and old hepatocytes, our sample size (ten single cells per age group) does not provide enough power to significantly distinguish potential differences between age groups. Interestingly, while not statistically significant, variance (from cell to bulk) of hepatocytes from the aged mice was higher than in the young animals (*P* = 0.148, one-tailed permutation test on the mean variance of each group) (Fig. 3a, b). Higher heterogeneity in hepatocytes compared with fibroblasts was confirmed when comparing the fraction of differentially methylated windows (DMWs) rather than comparing the variance (Fig. 3b; Additional file 1: Supplemental Experimental Procedures). Also in this case the DMW frequency was slightly higher among hepatocytes from the aged mice, with a profound increase in cell-to-cell variation.

To translate the observed variance (from cell to bulk) among hepatocytes into epivariation frequency, we then calculated the ratio between the number of altered CpG sites and the total number of CpGs overlapping between individual cells and the bulk (Additional file 1: Supplemental Experimental Procedures and Figure S3). Epivariation frequency in the hepatocytes appeared to be remarkably high, i.e., in the order of 3.3 % of all CpG sites analyzed. This is more than three orders of magnitude higher than the frequency of somatic DNA sequence mutations [11] and very similar to our previous promoter-based estimates [3].

Next, we explored whether heterogeneity of 5mC in mouse liver was dependent on specific genomic features. First we grouped all the hepatocytes together as we did not find a significant difference between young and old (Fig. 3a). To reduce bias due to coverage and sample size, each bin in each cell was down-sampled to 5 CpG counts per bin, irrespective of their presence in multiple reads at the same site or at multiple sites. After downsampling, we retained ten cells with the highest coverage for each bin, making the comparison of variation in methylation content homogeneous in all bins across the genome (Additional file 1: Supplemental Experimental Procedures). Our results indicate that 5mC heterogeneity is highly dependent on the genomic context and mostly a feature of non-functional sites. More specifically, 5mC variance between cells was higher than the genome average in repeats and transposons, whereas it was lower in functional sequences, such as CpG islands (CGIs) and promoter regions, 5′ untranslated regions (UTRs), exons, H3k36me3, and H3k4me3 (Fig. 3c), which is in keeping with previous work suggesting that hypermethylated regions are more error prone than hypomethylated ones [1, 2]. The exception appeared to be in the regions associated with histone H3 methylation at Lys4 (H3K4me1), the transcription-associated histone modification. Of note, H3K4me1 has been previously found to be associated with 5mC loss during aging [12].

Next, we focused on promoter 5mC heterogeneity, which appeared to depend on whether they were CGI or non-CGI promoters. Non-CGI promoters were found to be more variable than the CGI ones (Fig. 3c). In this respect, we can speculate that because non-CGI promoter genes are destined to change during development and adult life [13], they might be subjected to higher levels of fluctuations than the CGI ones.

## Conclusions

In recent years, extensive studies on epigenetic processes have revealed how complex genomes generate different cell types in a highly dynamic fashion. Once established, the epigenetic factors, such as DNA methylation and histone modification, need to be faithfully transmitted in cells during cell division or DNA damage repair to maintain cell identity. The large volume of epigenomic transactions and its continuous need for maintenance suggest a high chance of errors. However, virtually nothing is known on the epimosaicism within populations of seemingly identical cells. Herein, by employing a protocol for WGBS of single cells, we show that epivariation frequency in DNA methylation patterns is at least two orders of magnitude higher than somatic mutation frequencies [11, 14]. While to our knowledge this has never been directly analyzed in mammals, it is in accordance with reports suggesting that spontaneous transgenerational epigenetic changes in the *Arabidopsis thaliana* methylome are three orders of magnitude more frequent than DNA mutations [15, 16]. The observed high epivariation frequency in mouse liver is also in keeping with the previously observed relatively high levels of transmission infidelity of DNA methylation [1].

Our present data also indicate that 5mC heterogeneity level is dependent on genomic features, with non-functional sites, such as repeat elements and introns, mostly unstable and potentially functional sites, such as gene promoters, mostly stable. An interesting exception appeared to be the H3K4me1 epigenetic signature of active enhancers, which has been previously found associated with DNA methylation loss during aging [12]. These results are in accordance with those obtained by Smallwood et al. [5], who also showed the highest levels of heterogeneity in H3K4me1 when compared with other genomic features in single mouse embryonic stem cells. This result seems suggestive of a common heterogeneity signature among different cell types. Of note, we did not find any striking increase in 5mC heterogeneity with aging; we speculate that, at least in part, this could be due to the fact that epivariations are affected by both genetic and environmental factors, and we studied genetically identical mice reared under controlled conditions. It is also possible that the liver, being a reversible post-mitotic organ, under normal conditions has very little proliferative activity and, therefore, a limited chance of accumulating 5mC maintenance errors over time [1, 2, 5]. Finally, an age effect may actually be present, as suggested by a clear trend of a higher variance in the older animals, but simply obscured by the very high baseline of cell-to-cell variation.

The possible physiological effects of the high epigenome heterogeneity in adult liver remain unknown. It is conceivable that the observed epimosaicism reflects subtle but physiologically relevant variation within the hepatocyte population, similar to what has been postulated for neurons in the brain [17]. The mammalian liver performs a diverse range of critical functions for maintaining metabolic homeostasis (ranging from glucose regulation and lipid stores to blood detoxification). Therefore, a straightforward hypothesis is that the liver achieves this diversity through the collective behavior of heterogeneous hepatocytes operating in highly structured microenvironments. More specifically, hepatocytes with different epigenomes will have distinct molecular phenotypes and such heterogeneity, up to a certain extent, may be beneficial to the maintenance of organ functionality. However, because the observed epigenomic heterogeneity in mouse liver appeared to be fairly random, and while we did observe enrichment in sequences generally assumed to be non-functional, specific hotspots were not detected, it is probably more likely that epigenomic heterogeneity truly reflects errors. In this respect, what remains to be clarified is how much noise can be tolerated within an organ before its functionality would be impaired. We expect that ongoing development of single-cell technologies will allow noise effects to be tested by measuring cellular information status at multiple levels, i.e., genome, epigenome and transcriptome, of the same single cells [18–21]. Ongoing reduction in sequencing costs is likely to facilitate analysis of the large numbers of cells necessary for that purpose.

## Methods

### Animals

Three 4-month-old and three 26-month-old C57BL/6 male mice were obtained from the National Institute on Aging (NIA). All surgical procedures and experimental manipulations were approved by the Ethics Committee for Animal Experiments at the Albert Einstein College of Medicine. Experiments were conducted under the control of the Guidelines for Animal Experimentation. Animals were sacrificed by cervical dislocation.

### Ethics

The animals were sacrificed and primary cells were collected under the Institutional Animal Care and Use Committee (IACUC) of Albert Einstein College of Medicine protocol #20140308, “DNA Repair, Mutations and Cellular Aging” (approval 06 June 2014).

### Isolation of single mouse embryonic fibroblasts

Mouse embryonic fibroblasts (MEFs) were isolated from embryonic day 13.5 embryos of C57BL/6 mice as described [14]. All cultures were maintained in a 3 % O_2_ and 5 % CO_2_ atmosphere. After trypsinization, single MEFs were collected under an inverted microscope by hand-held capillaries, deposited in polymerase chain reaction (PCR) tubes and immediately frozen on dry ice and stored at −80 °C until needed or immediately bisulfite-converted.

### Isolation of single hepatocytes

Livers in six (three 4-month-old and three 26-month-old) C57BL/6 mice were perfused with collagenase following the protocol as described [22]. Single hepatocytes were stained with Hoechst 33342 and sorted using a MoFloXDP cell sorter (Beckman Coulter) into PCR tubes containing 5 μl of PBS, flash-frozen, and stored at −80 °C or immediately used for DNA methylation analysis.

### Genomic DNA extraction

DNA from MEF cultures or mouse liver was isolated by phenol/chloroform extraction, as described [11].

### Genomic DNA WGBS library preparation

DNA (100 ng) from bulk MEFs or hepatocytes was bisulfite-converted and subjected to library preparation using the Pico Methyl-Seq™ Library Prep Kit (Zymo) according to the instructions of the supplier. Libraries were assessed for quality using High-Sensitivity DNA chips on the Agilent Bioanalyzer and quantified with Qubit fluorometer. Libraries were sequenced on an Illumina HiSeq2500 (100-bp single-end sequencing).

### Single-cell lysis and WGBS library preparation

Single cells were lysed with 10 μl digestion buffer (Zymo) and 1 μl Proteinase K (Zymo) for 20 min at 50 °C in a total volume of 20 μl. The bisulfite conversion and library preparation were performed on cell lysates using the Pico Methyl-Seq™ Library Prep Kit (Zymo) according to the instructions of the supplier with some modifications. More specifically, as the first modification consisted of a reduction of the primer concentration in the pre-amplification step (20 μM) in order to avoid primer dimers in the final library. Subsequently, we introduced another modification at the amplification step: additional cycles were added to the amplification step and we therefore performed 11 cycles of PCR amplification in total. Libraries were assessed for quality using High-Sensitivity DNA chips on the Agilent Bioanalyzer. The quantity of each sequencing library was measured with a Qubit fluorometer. Libraries were sequenced on an Illumina HiSeq2500 (100-bp single-end sequencing).

### Sequencing data processing and analysis

Raw sequence data were subjected to quality control by FastQC v0.10.1 (http://www.bioinformatics.babraham.ac.uk/projects/fastqc/) and trimmed using trim galore v0.3.3 (http://www.bioinformatics.babraham.ac.uk/projects/trim_galore/) with default parameters except additional trimming of the first four and last two base pairs of a read due to abnormal GC content. Trimmed sequences were mapped to the mouse reference genome (mm9) using Bismark 0.10.0 with the alignment tool Bowtie2 2.1.0. Sequence duplicates were further removed and single CpG methylation was called using Bismark [23]. A summary of data processing is shown in Additional file 1: Table S1.

To estimate CpG methylation variations, a sliding window of 3 kb in size and 600 bp in step size was used to subdivide the genome, similar to Smallwood et al. [5]. Windows covering at least 5 CpGs were used in the analysis (Fig. 1c; Additional file 1: Figure S1). The methylation frequency of a window in one sample was estimated based on a binomial distribution.

Heterogeneity levels were estimated in two ways”: (1) global difference between a cell and its bulk; and (2) local difference between cell–bulk pairs in each window. In both, heterogeneity level is quantified using a weighted variance value, for which mean methylation frequency is approximated using the corresponding bulk. Multiple downsamplings were performed to access potential noise due to technical artifacts (Fig. 3a). Annotations of genomic features were obtained from multiple resources (Additional file 1: Table S3).

Of note, our definition of variance in genomic features is slightly different from Smallwood et al [5]. They plotted the lower bounds of the 95 % confidence interval and we plotted the estimated mean. Additionally, raw variance value is biased by sequencing depth. For example, if the methylation level of two 5mCs are both 0.5 but the sequencing depths are 3× and 20× separately, there will be a systematic bias comparing sequencing depth at 3× (most likely 0.67 or 0.33) of 5mC and 20× (close to 0.5) of the other. We therefore downsampled the data to reach the same sequencing depth in all genomic regions. The downsampling provides a less biased comparison at the cost of more noise. Thus, our reported variances are less quantitatively different than those in Smallwood et al. [5] but more directly comparable.

Finally, epivariation was defined as methylation difference between a single cell and its bulk at a single CpG site. To call an epivariation at a 5mC, we required (1) a sequencing depth at the 5mC site larger than 5 in both single cell and bulk; (2) more than 90 % of the reads in bulk showing the same methylation pattern (either methylation or unmethylation); and (3) more than three reads in the cell indicating a different methylation pattern than the bulk. Epivariation frequency is stable even with slight changes to the above criteria (Additional file 1: Figure S3). Further details of data analysis are described in Additional file 1: Supplemental Experimental Procedures.

## Additional file

Additional file 1: Supplementary materials. The supplementary materials include Figures S1–S3, Tables S1–S3, and Supplementary Experimental Procedures. (PDF 473 kb)
