# Supplementary material for: Single-cell genome-wide bisulfite sequencing uncovers extensive heterogeneity in the mouse liver methylome
Source: Genome Biol. 2016 Jul 5;17:150. doi: 10.1186/s13059-016-1011-3 (PMC4934005; doi:10.1186/s13059-016-1011-3)
Supplement: Supplementary file 1 — Supplementary materials. The supplementary materials include Figures S1–S3, Tables S1–S3, and Supplementary Experimental Procedures. (PDF 473 kb) [file 13059_2016_1011_MOESM1_ESM.pdf]

# Supplemental Information

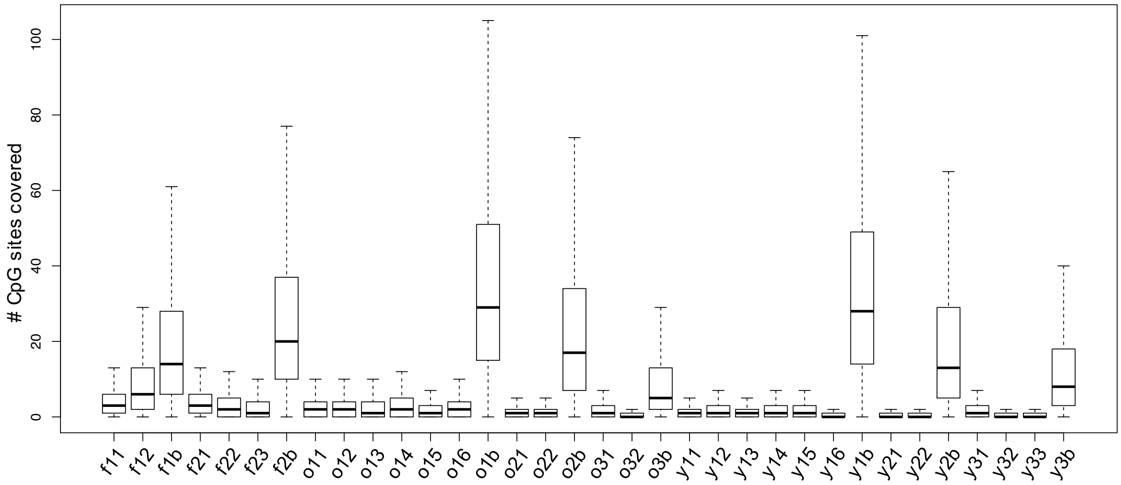


**Figure S1. Sequencing coverage on CpGs in 3kb sliding windows.** Single fibroblasts - f11, f12, f21, f22, f23; fibroblast bulks - f1b, f2b; hepatocytes old - o11 to o16, o21, o22, o31, o32; bulk hepatocyte old - o1b, o2b, o3b; hepatocytes young - y11 to y16, y21, y22, y31, y32, y33; bulk hepatocytes young - y1b, y2b, y3b.

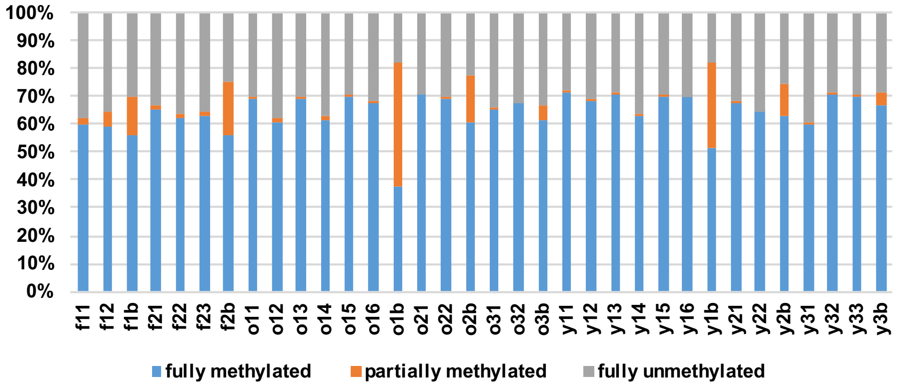


**Figure S2. Distribution of single CpG methylation status in single cells and bulks**

a)

b)

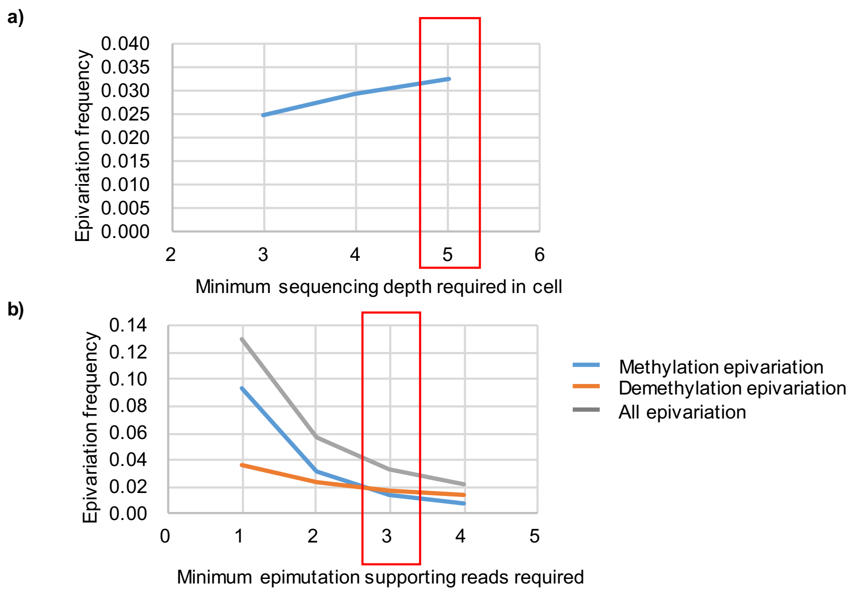


**Figure S3. Criteria for epivariation calls. a)** Effect of minimum sequencing depth (x-axis) on epivariation frequency estimation (y-axis). **b)** Effect of minimum epivariation supporting reads (x-axis) on epivariation frequency estimation (y-axis). Red boxes indicated final criteria.

**Table S1. Bisulfite sequencing data processing summary.**

| Sample* | # raw reads | Mapping efficiency | Bisulfite conversion rate** | Sequence duplication level*** | # CpG (depth>=1x) | Age (month) | Cell/bulk | cell type |
|---|---|---|---|---|---|---|---|---|
| f11 | 106,231,336 | 42.3% | 98.7% | 75.3% | 3,785,324 | - | cell | MEF |
| f12 | 110,901,598 | 51.1% | 98.7% | 56.0% | 8,507,920 | - | cell | MEF |
| f1b | 132,443,151 | 58.0% | 99.0% | 16.8% | 17,826,778 | - | bulk | MEF |
| y21 | 51,858,580 | 38.6% | 98.8% | 92.4% | 825,218 | 4 | cell | Hepatocyte |
| y22 | 66,424,232 | 29.0% | 97.6% | 92.8% | 456,387 | 4 | cell | Hepatocyte |
| y2b | 152,888,716 | 60.5% | 98.9% | 25.9% | 19,050,595 | 4 | bulk | Hepatocyte |
| y31 | 31,913,863 | 26.8% | 98.6% | 70.4% | 1,890,962 | 4 | cell | Hepatocyte |
| y32 | 43,059,862 | 13.8% | 98.8% | 84.3% | 576,677 | 4 | cell | Hepatocyte |
| y33 | 26,410,825 | 29.4% | 98.9% | 88.8% | 461,319 | 4 | cell | Hepatocyte |
| y3b | 57,982,762 | 60.1% | 99.0% | 8.5% | 11,740,589 | 4 | bulk | Hepatocyte |
| o21 | 56,089,254 | 24.4% | 98.7% | 82.2% | 1,425,722 | 26 | cell | Hepatocyte |
| o22 | 50,744,157 | 22.5% | 98.8% | 79.2% | 1,437,004 | 26 | cell | Hepatocyte |
| o2b | 156,376,935 | 55.9% | 98.9% | 32.7% | 22,384,321 | 26 | bulk | Hepatocyte |
| o31 | 58,584,746 | 24.0% | 98.4% | 81.8% | 1,713,053 | 26 | cell | Hepatocyte |
| o32 | 50,378,180 | 25.3% | 98.6% | 82.0% | 831,435 | 26 | cell | Hepatocyte |
| o3b | 38,087,858 | 60.3% | 98.9% | 7.7% | 8,869,698 | 26 | bulk | Hepatocyte |
| f21 | 13,121,264 | 53.2% | 99.1% | 24.8% | 4081273 | - | cell | MEF |
| f22 | 11,189,137 | 49.6% | 99.0% | 25.6% | 3307076 | - | cell | MEF |
| f23 | 33,944,267 | 37.9% | 98.9% | 73.8% | 2363888 | - | cell | MEF |
| f2b | 147,930,511 | 44.7% | 99.0% | 10.7% | 24598670 | - | bulk | MEF |
| o11 | 27,097,243 | 44.5% | 98.7% | 67.8% | 2596479 | 26 | cell | Hepatocyte |
| o12 | 41,395,901 | 22.3% | 98.5% | 58.0% | 2666715 | 26 | cell | Hepatocyte |
| o13 | 78,054,583 | 28.0% | 97.9% | 80.4% | 2410838 | 26 | cell | Hepatocyte |
| o14 | 57,368,890 | 46.8% | 98.7% | 80.1% | 3337756 | 26 | cell | Hepatocyte |
| o15 | 66,121,632 | 42.8% | 98.5% | 86.2% | 2163080 | 26 | cell | Hepatocyte |
| o16 | 28,019,317 | 39.2% | 98.9% | 64.3% | 2767543 | 26 | cell | Hepatocyte |
| o1b | 478,304,583 | 56.8% | 99.1% | 30.1% | 33654426 | 26 | bulk | Hepatocyte |
| y11 | 41,893,787 | 18.3% | 98.0% | 69.4% | 1506708 | 4 | cell | Hepatocyte |
| y12 | 79,871,545 | 24.2% | 97.8% | 83.1% | 1659713 | 4 | cell | Hepatocyte |
| y13 | 42,767,824 | 25.3% | 98.1% | 76.1% | 1590036 | 4 | cell | Hepatocyte |
| y14 | 52,037,967 | 14.4% | 98.0% | 61.1% | 2014092 | 4 | cell | Hepatocyte |
| y15 | 56,839,977 | 38.3% | 98.4% | 83.4% | 2005993 | 4 | cell | Hepatocyte |
| y16 | 51,075,606 | 27.0% | 98.1% | 87.1% | 902484 | 4 | cell | Hepatocyte |
| y1b | 369,426,587 | 55.8% | 99.0% | 23.4% | 32332245 | 4 | bulk | Hepatocyte |

* fab/oab/yab - a indicates mouse individual a; b indicates cell id or bulk.

** Bisulfite conversion rate is estimated as the ratio between the number of non-CpG methylations and total non-CpGs

*** Duplicated sequences, mostly a result of PCR, were marked by bismark and removed.

**Table S2. Comparision between our and previous single cell whole-genome bisulfite sequencing methods.**

| Method | Species | Single end or paired end | Analyzable reads | Bisulfite conversion rate | Unique CpGs Covered |
|---|---|---|---|---|---|
| Gravina | mouse | single-end | 4,132,144 | 98.51% | 2,203,258 |
| Farlik* | human | paired-end | 687,423 | >99% | 1,261,269 |
| Farlik* | mouse | paired-end | 719,999 | >99% | 1,028,520 |
| Smallwood** | mouse | paired-end | 3,393,033 | 97.53% | 3,891,832 |

*Smallwood SA, Lee HJ, Angermueller C, Krueger F, Saadeh H, Peat J, et al. Nat Methods. 2014 Aug;11(8):817-20.

**Farlik M, Sheffield NC, Nuzzo A, Datlinger P, Schönegger A, Klughammer J, Bock C. Cell Rep. 2015 Mar 3;10(8):1386-97.

**Table S3. Resource of genome annotations.**

| Annotation | Counts | Resource | Defined by or as |
|---|---|---|---|
| CpG island (repeat masked version) | 16,027 | UCSC | Irizarry, RA. Et al., *Nat Genet* , 2009 |
| CpG island shore | 32,053 | - | 2kb flanking CpG island |
| Protein coding gene transctiptionsites | 76,835 | Ensembl Biomart | Ensembl Biomart |
| Protein coding gene exon | 383,868 | Ensembl Biomart | Ensembl Biomart |
| Protein coding gene intron (consensus) | 207,353 | Based on gene and exon from Ensembl Biomart | - |
| Protein coding gene 5' utr | 61,497 | Ensembl Biomart | Ensembl Biomart |
| Protein coding gene 3' utr | 56,117 | Ensembl Biomart | Ensembl Biomart |
| Promoter | 76,835 | - | TSS to its 2kb upstream |
| Repeat DNA transposon | 158,329 | repeatMask | repeatMask |
| Repeat LINE | 969,721 | repeatMask | repeatMask |
| Repeat LTR | 854,046 | repeatMask | repeatMask |
| Repeat SINE | 1,520,027 | repeatMask | repeatMask |
| Repeat simple repeat | 1,062,130 | repeatMask | repeatMask |
| Repeat other | 433,821 | repeatMask | repeatMask |
| H3K27ac | 38,492 | ENCODE, ID:ENCSR000CDH | ENCODE |
| H3K27me3 | 33,402 | ENCODE, ID:ENCSR000CEN | ENCODE |
| H3K36me3 | 88,353 | ENCODE, ID:ENCSR000CEO | ENCODE |
| H3K4me1 | 77,192 | ENCODE, ID:ENCSR000CAO | ENCODE |
| H3K4me3 | 16,888 | ENCODE, ID:ENCSR000CAP | ENCODE |
| H3K79me2 | 68,593 | ENCODE, ID:ENCSR000CEP | ENCODE |
| H3K9ac | 29,230 | ENCODE, ID:ENCSR000CEQ | ENCODE |
| Liver specific genes | 58 | Lin, S. et al., *PNAS* , 2014 | Lin, S. et al., *PNAS* , 2014 |

## A. Modeling methylation frequency using sliding windows

To quantify methylation frequency, we subdivided the genome using sliding windows of 3 kb in size and 600 bp in step size. Methylation counts of single CpGs within a window were pooled together. Windows with at least 5 CpGs covered are used in following analysis. Methylation frequency of a window i in sample (single cell or bulk) j was modeled as a binomial distribution. So methylation frequency is given as using Laplacian smoothing to avoid unreasonable 0 or 1 estimations when methylation count in a window is small,

$$\hat{m}_{i,j} = \frac{c_{i,j}^{+} + 1}{c_{i,j}^{+} + c_{i,j}^{-} + 2} \quad (1)$$

Where $c_{i,j}^{+}$ and $c_{i,j}^{-}$ are methylated counts and unmethylated counts separately. And the standard error is given as,

$$se_{i,j} = \sqrt{\frac{\hat{m}_{i,j} \cdot (1 - \hat{m}_{i,j})}{n_{i,j}}} \quad (2)$$

Where $n_{i,j}$ is the sum of $c_{i,j}^{+}$ and $c_{i,j}^{-}$.

## B. Estimating heterogeneity level

To quantify heterogeneity level, we first paired each cell with its bulk (in the following text we refer it to "pairs"), and then used variance value and made two estimations – 1) global difference across all windows for one cell and bulk pair to estimate the difference between the cell and its corresponding bulk; 2) local variance for one window across all cells to estimate the heterogeneity level within the window. Both estimations were based on the following.

Considering the single cell – bulk data structure, we first gave weight to each single cell – bulk pair p at window i,

$$se_{i,p} = \sqrt{se_{i,c}^{2} + se_{i,b}^{2}} \quad (3)$$

$$w_{i,p} = se_{i,p}^{-2} \quad (4)$$

Where c refers to a single cell and b refers to its corresponding bulk.

In the first estimation, difference between one cell and its bulk is,

$$\hat{v}_c = \frac{\sum_i w_{i,p}}{(\sum_i w_{i,p})^2 - \sum_i w_{i,p}^2} \times \sum_i \left( w_{i,p} \times \left( \hat{m}_{i,pc} - \hat{m}_{i,pb} \right)^2 \right) \quad (5)$$

Where $\hat{m}_{i,pc}$ and $\hat{m}_{i,pb}$ refers to the estimated methylation frequency from the single cell c and the bulk b of the pair p separately. By this definition (equation 5), we quantified the difference between one cell to its bulk over all the genome.

To rule out a concern that $\hat{v}_c$, which we detected were caused by artifacts of difference in sequencing depth between a single cell and its bulk, we estimated the artificial heterogeneity level $\hat{v}_{noise}$ caused only by the above concern using a downsampling as the following. We first downsampled the bulk data to the cell level. Specifically, we randomly selected x number of methylation counts over the whole genome from bulk, where x refers to the number of methylation counts we obtained from a single cell in whole genome. Then, we calculated $\hat{v}_{noise}$ using equation (5) and the downsampling were performed 20 times for each cell. **Figure 3a** shows that $\hat{v}_c$ is significantly higher than $\hat{v}_{noise}$ for all cells. This indicates that difference between cell and bulk we observed is not due to the above technical concerns.

Similar to 1), cell-to-cell variance in window i is,

$$\hat{v}_i = \frac{\sum_p w_{i,p}}{(\sum_p w_{i,p})^2 - \sum_p w_{i,p}^2} \times \sum_p \left( w_{i,p} \times \left( \hat{m}_{i,pc} - \hat{m}_{i,pb} \right)^2 \right) \quad (6)$$

To minimize potential bias caused by sequencing depth and coverage, in $\hat{v}_i$ estimation, we downsampled methylation counts to a same coverage level that for each window and each sample we have 5 counts in a single cell and 20 in the corresponding bulk. Variance value are approximate as the variance values from the downsampling. Additionally, considering the data structure – 21 single hepatocytes-bulk pairs, we included windows with at least 10 pairs in each group passed criteria for the variance estimation (283,726 windows, about 11% of the genome), and the estimation is based on the top 10 best covered pairs in each group (**Figure 3c**). By this definition (equation 6), we quantified the variations of a specific region (window) among a group of single cells.

## C. Defining differentially methylated windows

We used the following test statistic to identify differentially methylated windows, i, between a single cell and its corresponding bulk,

$$s_{i,p} = \frac{\hat{m}_{i,c} - \hat{m}_{i,b}}{se_{i,p}} \quad (7)$$

where $se_{i,p}$ is the pooled standard error of a single cell – bulk pair defined by (3). For windows passing all of the following criteria,

$$\hat{m}_{i,c} \cdot n_{i,c} > 5 \quad (8)$$

$$(1 - \hat{m}_{i,c}) \cdot n_{i,c} > 5 \quad (9)$$

$$\hat{m}_{i,\mathrm{b}} \cdot n_{i,\mathrm{b}} > 5 \quad (10)$$

$$(1 - \hat{m}_{i,\mathrm{b}}) \cdot n_{i,\mathrm{b}} > 5 \quad (11)$$

we applied two-sided z-test, and for windows don't pass the criteria, t-test. Thus, p values and confidence intervals (CIs) for windows were obtained. Windows with p value less than 0.0001 and the absolute value of two bonds confidence interval larger than 0.1, were defined as differentially methylated windows.

## D. Estimating epivariation frequency

We define epivariation as methylation difference between a single cell and its bulk at a single CpG site, specifically, mostly (≥90%) methylated or unmethylated in bulk and multiple supporting reads (≥3) in a cell supporting the alternative methylation statues (in both cell and bulk requiring sequencing depth ≥ 5). Results under different depth and supporting reads criteria were presented in **Figure S3**. Thus epivariation frequency was calculated as a ratio between number of epivariation and CpG sites passed the depth criteria.

## E. Others

Circos plot (**Figure 1a**) was generated using R package OmicCircos (Hu et al., 2014).

## Supplemental References

Hu, Y., Yan, C., Hsu, C.H., Chen, Q.R., Niu, K., Komatsoulis, G.A., and Meerzaman, D. (2014). OmicCircos: A Simple-to-Use R Package for the Circular Visualization of Multidimensional Omics Data. Cancer informatics *13*, 13-20.
